# Exonuclease III Can Efficiently Cleave Linear Single-Stranded DNA: Reshaping Its Experimental Applications in Biosensors

**DOI:** 10.3390/bios13060581

**Published:** 2023-05-26

**Authors:** Yi Shen, Haoyu Yuan, Zixuan Guo, Xiu-Qing Li, Zhiqing Yang, Chengli Zong

**Affiliations:** 1 State Key Laboratory of Marine Resource Utilization in South China Sea, Key Laboratory of Tropical Biological Resources of Ministry of Education, School of Pharmaceutical Sciences, Hainan University, Haikou 570228, China; 2 Fredericton Research and Development Centre, Agriculture and Agri-Food Canada, Fredericton, NB E3B 4Z7, Canada; 3 NutraHealth Products and Technologies Inc., Fredericton, NB E3B 6J5, Canada; 4 Rizhao Science and Technology Innovation Service Center, Rizhao 276825, China

**Keywords:** exonuclease III, target recycling amplification assay, exonuclease I

## Abstract

Exonuclease III (Exo III) has been generally used as a double-stranded DNA (dsDNA)-specific exonuclease that does not degrade single-stranded DNA (ssDNA). Here, we demonstrate that Exo III at concentrations above 0.1 unit/μL can efficiently digest linear ssDNA. Moreover, the dsDNA specificity of Exo III is the foundation of many DNA target recycling amplification (TRA) assays. We demonstrate that with 0.3 and 0.5 unit/μL Exo III, the degradation of an ssDNA probe, free or fixed on a solid surface, was not discernibly different, regardless of the presence or absence of target ssDNA, indicating that Exo III concentration is critical in TRA assays. The study has expanded the Exo III substrate scope from dsDNA to both dsDNA and ssDNA, which will reshape its experimental applications.

## 1. Introduction

Traditionally, exonuclease III (Exo III) is used as a dsDNA-specific exonuclease that can catalyze the stepwise removal of mononucleotides from 3′-hydroxyl termini of dsDNA [1]. It favorably cleaves 3′ termini of linear dsDNA (L-dsDNA) with 5′ overhangs or blunt ends and 3′ overhangs containing fewer than four bases [2,3]. Exonuclease III can degrade from a nick on a circular dsDNA in the 3′ to 5′ direction, producing single-stranded circular DNA [4]. Exo III is a distributive enzyme that frequently dissociates during digestion [5]. However, dsDNA with 4-base or longer 3′-protruding termini was resistant to Exo III degradation in earlier reports [6]. Many reports have asserted that Exo III will not degrade ssDNA [7,8]. Inspired by this unique feature, Exo III has been widely used for target recycling amplification (TRA) [9,10,11,12]. Target DNA with protruding 3′-termini can form a hybrid with a single-stranded DNA probe, which can be selectively digested to generate a signal and release the target DNA to re-hybridize with another probe to initiate the subsequent cycling cleavage process.

In our efforts to develop next-generation Exo III-assisted TRA, we were surprised to discover that Exo III could efficiently degrade ssDNA, contradicting traditional Exo III substrate specificity. To evaluate the performance, we studied a few critical aspects for Exo III ssDNA cleavage activity, compared the ssDNA cleavage activity of Exo I and III, and assessed its performance in TRA.

## 2. Materials and Methods

### 2.1. Materials

Exo III was purchased from New England Biolabs Co., Ltd. Beijng, China (LOT: 10100276); Exo I was purchased from Sangon Biotech Co., Ltd. Shanghai China (LOT: F815DA0004). Exo III reaction buffer: 10 mM Tris-HCl, 10 mM MgCl_2_, 1 mM DTT, pH 7.0; Exo I reaction buffer: 6.7 mM glycine-KOH (pH 9.5 at 25 °C), 6.7 mM MgCl_2_, 1 mM DTT. All deoxyribonucleic acids were fabricated by Sangon Biotech Co., Ltd. Shanghai China and their sequences are listed in Table S1.

### 2.2. Fluorescence Detection of ssDNA FQ Reporter Digestion by Exonuclease III

Fluorescence plate reader (BioTek H1 microplate reader) detection settings: temperature: 37 °C; λ_ex_: 492 nm; λ_em_: 520 nm; fluorescence measurements were taken every 3 s for up to 30 s. First, 10 μL of Exo III was quickly added to a 96-well microplate containing the 90 μL FQ ssDNA reporter (final concentrations of 0.05, 0.01, 0.03, 0.1, 0.2, 0.3, 0.5, and 1 unit/μL). Then, the fluorescence was recorded immediately. The background signal was obtained by replacing Exo III with the reaction buffer. FQ ssDNA reporter and Exo III were diluted by the Exo III reaction buffer.

### 2.3. Exo III-Assisted Target Recycling Amplification with L-ssDNA Probe Free in Buffer

Detection settings: temperature: 37 °C; λ_ex_: 492 nm; λ_em_: 520 nm; fluorescence measurements were taken every 10 s for up to 30 min. First, 10 μL FQ probe was added into a 96-well microplate containing 70 μL reaction buffer. Then, 10 μL of different concentrations of target DNA was added into the microplate and incubated for 10 min at 37 °C. The fluorescence was recorded by a BioTek H1 microplate reader. Then, 10 μL of Exo III was quickly added into the above 96-well microplate (the final target DNA concentrations were 100 nM, 10 nM, 1 nM; the final Exo III concentration was 0.3 unit/μL) and the fluorescence was recorded immediately. The background signal was obtained by replacing Exo III with the reaction buffer.

### 2.4. Exonuclease III Digestion of dsDNA with 3′ 4-nt Overhang dsDNA or Blunt-Ended dsDNA

First, Exo III reaction buffer containing 500 nM ssDNA1 and 500 nM ssDNA2 was heated to 95 °C for 5 min. After cooling down to room temperature for 30 min, the formed 3′ 4-nt overhang dsDNA was digested by 0.2 unit/μL Exo III at 37 °C for 1 h. The mixture was verified by 15% PAGE.

The blunt-ended dsDNA was prepared with ssDNA1 and ssDNA3. The rest of the experimental procedures were the same as those above.

## 3. Results and Discussion

### 3.1. Exo III ssDNA Cleavage Property

**Figure 1:**
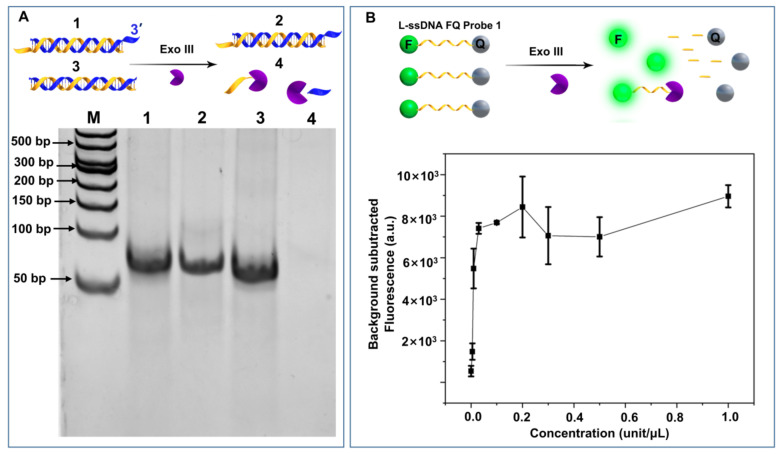
Impact of Exo III concentration on ssDNA cleavage activity. (**A**) Exo III cleaves blunt-end dsDNA and leaves dsDNA 3′-overhang dsDNA intact for 1 h (1: 500 nM dsDNA 3′-4 nt overhang; 2: 500 nM dsDNA 3′-4 nt overhang + 0.2 unit/μL Exo III; 3: 500 nM dsDNA; 4: 500 nM dsDNA + 0.2 unit/μL Exo III). (**B**) Exo III cleaves ssDNA FQ probe (1 h) at various concentrations (serial dilutions including: 1, 0.5, 0.3, 0.2, 0.1, 0.03, 0.01, 0.005, and 0 unit/μL).

The ability of Exo III to digest blunt-ended dsDNA, but not 3′ 4-nt overhang dsDNA, was confirmed by a polyacrylamide gel electrophoresis (PAGE) study (Figure 1A, lanes 4 and 3). However, we were surprised to find 0.1 unit/μL Exo III from New England Biolabs Ltd. was capable of efficiently cleaving an ssDNA fluorescence quencher (FQ) reporter. A series of Exo III were incubated with a linear ssDNA FQ reporter (12 nt) to investigate the correlation between concentration and ssDNA cleavage activity. As shown in Figure 1B, the fluorescence plateaued at concentrations from 0.03 unit/μL to 1 unit/μL. It should be noted that many research groups employ around 1 unit/μL Exo III to specifically cleave dsDNA. Those results create a dilemma: if Exo III can digest ssDNA, how can the ssDNA target survive the target recycling amplification, which commonly employs Exo III to cleave the ssDNA FQ reporter to release and recycle ssDNA target from its hybrid with the FQ reporter. To answer those questions, we decided to evaluate Exo III’s ssDNA cleavage performance.

### 3.2. Comparison of Exo III and Exo I ssDNA Cleavage Property

**Figure 2:**
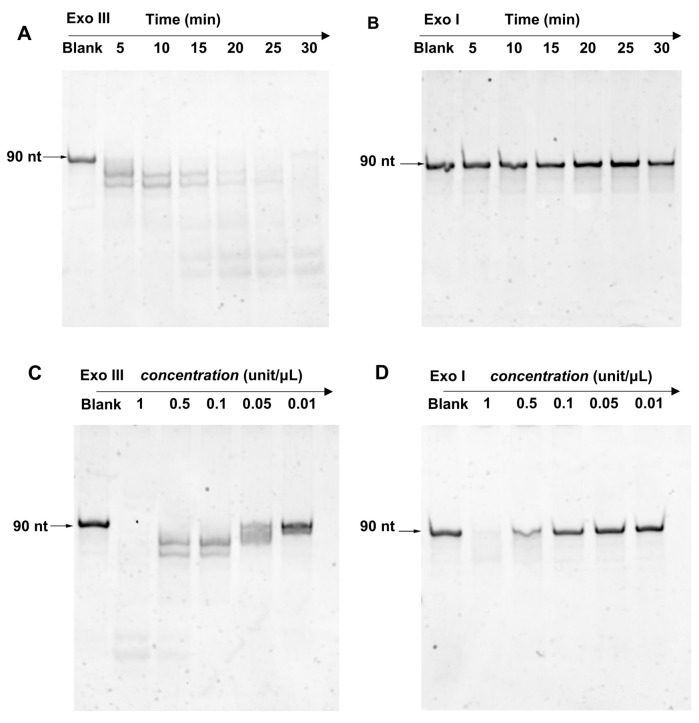
PAGE analysis of 90 nt ssDNA (2 uM) treated with (**A**) Exo III and (**B**) Exo I (0.1 unit/μL) for different times (5 to 30 min), PAGE analysis of 90 nt ssDNA (2 uM) treated with various concentrations of (**C**) Exo III and (**D**) Exo I (0.01 to 1 unit/μL) for 5 min.

First, we compared the ssDNA cleavage activity of Exo III and Exo I. Exo I is a classic 3′ → 5′ ssDNA-specific exonuclease. As shown in Figure 2A, a 90 nt ssDNA reporter was fully digested by 0.1 unit/μL Exo III within 30 min. However, at the same concentration, Exo I could barely digest the reporter within the tested time frame (Figure 2B). By increasing the concentration to at least 0.5 unit/μL (Figure 2D), digestion was observed. In contrast, Exo III displayed cleavage activity at concentrations as low as 0.05 unit/μL (Figure 2C). These data suggested that at concentrations above 0.05 unit/μL, Exo III cleaves ssDNA more efficiently than Exo I.

### 3.3. Exo III ssDNA Substrate in Target Recycling Assays

To increase the signal to target ratio for nucleic acid detection methods, TRA is commonly used to amplify the signal. Exo III was employed to selectively digest the FQ-probe from the 3′ end of the dsDNA hybrid to release the target ssDNA, because it has been viewed as a dsDNA-specific enzyme. The released target ssDNA will then hybridize with another FQ probe to repeat the process, amplifying the fluorescence signal. However, our data indicated that ssDNA can be efficiently cleaved at a concentration range between 0.1 to 1 unit per μL Exo III, which covers most of the reported TRA Exo III concentrations.

**Figure 3:**
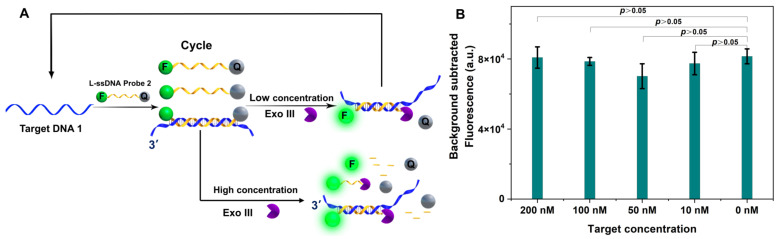
(**A**) The role of different concentrations of Exo III-assisted target recycling amplification. (**B**) The fluorescence intensity of different concentrations of target DNA with a high concentration of Exo III-assisted target recycling amplification (*n* = 3 technical replicates, bars represent mean ± SEM).

To resolve this puzzle, we explored the ssDNA cleavage activity of two types of Exo III-assisted TRA with an L-ssDNA probe free in the reaction buffer or fixed on a solid surface. First, target DNA1 could hybridize with the L-ssDNA FQ probe 3 (sequence in Table S1) in solution (Figure 3A). The L-ssDNA FQ probe with recessed 3′-termini in dsDNA could be selectively digested by Exo III to generate a fluorescence signal. In our assay, different concentrations of target DNA were used (200 nM, 100 nM, 10 nM, and 1 nM) in the Exo III (0.3 unit/μL)-assisted TCA. However, the fluorescence intensity was not discernibly different with or without target DNA (Figure 3B). PAGE (Figure S1 in Supplementary Materials) was used to further confirm the fluorescence study results. The data indicated that a high concentration of Exo III could cleave both target DNA1 and L-ssDNA probe 2.

**Figure 4:**
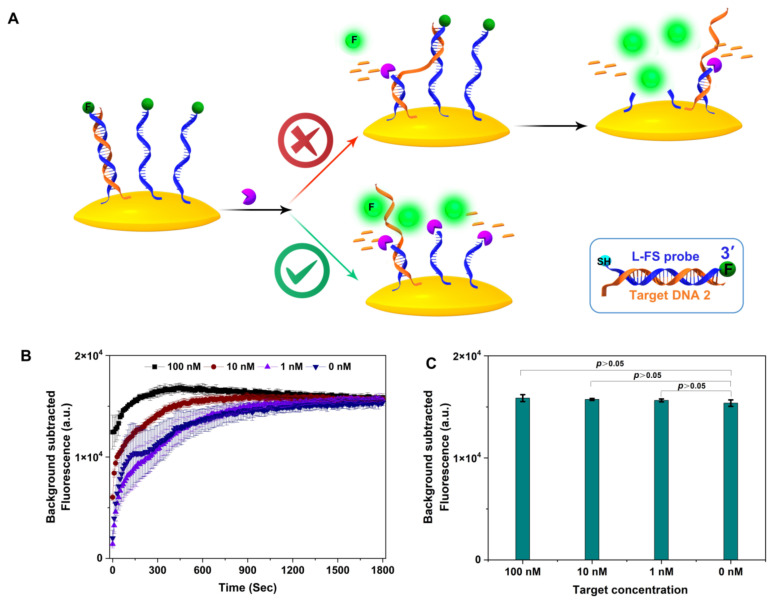
(**A**) The scheme of Exo III-assisted DNA walking and limitation of exonuclease III in recycling amplification, (**B**) cleavage kinetics of Exo III-assisted DNA walking for different concentrations of target DNA, (**C**) the fluorescence intensity of different concentrations of target DNA in Exo III-assisted DNA walking at 30 min (*n* = 3 technical replicates, bars represent mean ± SEM).

Next, we tested Exo III-assisted target recycling amplification with an L-ssDNA probe fixed on the surface of the gold nanoparticles (AuNPs). As shown in Figure 4, the thiol-modified fluorescent linear-FAM-labeled ssDNA probe (L-FS probe; for sequence, see Table S1) was fixed on the AuNPs, the fluorescence of which was quenched. Theoretically, target DNA partially hybridized L-FS probe with recessed 3-termini in dsDNA can be selectively digested by Exo III to generate a fluorescence signal, and the released target ssDNA can walk on the AuNPs surface via hybridizing with neighboring DNA probes until probes are completely digested (upper scheme in Figure 4A). A similar concept has been applied on the cell surface [13], magnetic bead surface [14], and electrode surface [15].

Exo III concentrations vary significantly across literature from 10^−6^ to 1 unit/μL. We selected 0.5 unit/μL Exo III and various concentrations of target DNA (100 nM, 10 nM, and 1 nM) to perform the Exo III-assisted target DNA walking assay. Cleavage kinetics indicated the cutting rate correlated with the concentration of target DNA. This makes sense because Exo III cleaves the hybrid dsDNA of target ssDNA and ssDNA probes faster than ssDNA [16]. The final fluorescence intensities showed no difference between target concentrations. Moreover, the fluorescence signal at 30 min was not discernibly different with or without target DNA (Figure 4C). The results confirmed that at a concentration of 0.5 unit/μL, Exo III can cleave the FQ probe fixed on a solid surface. It should be noted that Qu et al., reported the same assay using very a low concentration of Exo III (10^−6^ unit/μL) and the total reaction time was 12 h [11].

## 4. Conclusions

In summary, Exo III has been mostly used as a dsDNA-specific nuclease. However, some researchers have observed that Exo III can cleave ssDNA [17], which has been considered undesirable and to contribute to assay background [18,19]. However, to our best knowledge, Exo III ssDNA cleavage activity has been overlooked and detailed studies have not been reported. We demonstrate that Exo III can efficiently cleave ssDNA at concentrations above 0.1 unit/μL. By increasing Exo III concentration from 0.025 to 0.125 unit/μL, dsDNA and ssDNA were both completely digested, indicating the differential specificity of Exo III for dsDNA and ssDNA is less than a factor of five.

The dsDNA specificity of Exo III is the foundation of many TRA assays. However, our data indicate that at a concentration of 0.3 or 0.5 unit/μL, Exo III can efficiently cleave the ssDNA probe regardless of the presence or absence of target DNA. This evidence proves that the dsDNA specificity of Exo III is overrated by researchers. The study expanded Exo III substrate scope from dsDNA to both dsDNA and ssDNA, which may reshape its experimental applications.

## Data Availability

Not applicable.

## Supplementary Materials

The following supporting information can be downloaded at: https://www.mdpi.com/article/10.3390/bios13060581/s1, Figure S1: Analysis of Exo III-assisted free L-ssDNA probe in RCA reaction buffer by 15% PAGE. (A) The role of different concentrations of Exo III-assisted target recycling amplification; (B) Gel image of analysis of different concentrations of target DNA with a high concentration of Exo III-assisted target recycling amplification; Table S1: The sequences of deoxyribonucleic acids.
