# Supplementary material for: Exonuclease III Can Efficiently Cleave Linear Single-Stranded DNA: Reshaping Its Experimental Applications in Biosensors
Source: Biosensors (Basel). 2023 May 26;13(6):581. doi: 10.3390/bios13060581 (PMC10295840; doi:10.3390/bios13060581)
Supplement: Supplementary file 1 [file biosensors-13-00581-s001.zip › biosensors-2345103-supplementary [Updated].pdf]

Supporting Information for:

Exonuclease III Can Efficiently Cleave Linear Single-Stranded DNA: Reshaping Its Experimental Applications in Biosensors

Yi Shen [1,†], Haoyu Yuan [1,†], Zixuan Guo [1], Xiu-Qing Li [2,3], Zhiqing Yang [1,4,*] and Chengli Zong [1,*]

[1] State Key Laboratory of Marine Resource Utilization in South China Sea, Key Laboratory of Tropical Biological Resources of Ministry of Education, School of Pharmaceutical Sciences, Hainan University, Haikou 570228, China
[2] Fredericton Research and Development Centre, Agriculture and Agri-Food Canada, Fredericton, NB E3B 4Z7, Canada
[3] NutraHealth Products and Technologies Inc., Fredericton, NB E3B 6J5, Canada
[4] Rizhao Science and Technology Innovation Service Center, Rizhao 276825, China
* Correspondence: yangzhiqing@hainanu.edu.cn (Z.Y.); chengli.zong@hainanu.edu.cn (C.Z.)
† These authors contributed equally to this work.

Experimental Procedures

The Exonuclease digestion of 90 nt ssDNA with different timings

First, the 90 ssDNA and Exo III were mixed in the Exo III reaction buffer to reach final concentrations of 0.2 unit/μL and 1 μM, respectively. After digestion for 5 min, 10 min, 15 min, 20 min, 25 min and 30 min at 37 ^{o}C, 20 μL of the solution was taken out to 200 μL centrifuge tubes and quickly treated with boiling water for 20 min to inactivate Exo III, which was investigated by 15% PAGE.

Similar procedures were followed for Exo I (0.2 unit/μL) digestion of 90 nt ssDNA in Exo I reaction buffer.

Exo III-assisted target recycling amplification with L-ssDNA probe fixed on AuNPs surface.

The oligonucleotides-AuNP conjugates were prepared according to a previous report [1]. Briefly, thiol-modified fluorescence oligonucleotides L-FS probe (final concentration was 10 μM) were added to an aqueous solution of the prepared AuNPs (final concentration is 10 nM). After incubation for 12 hours, solutions of sodium dodecylsulphate (SDS), phosphate buffer (pH = 7.4), and NaCl were added in six portions at 80 minutes interval over eight hours to achieve final concentrations of 0.1%, 10 mM and 0.3 M, respectively. After being shaken for 12h, the AuNPs were washed by the reaction buffer for three successive rounds of centrifugation (12,000 rcf, 20 min) and the supernatant of the first centrifugation was collected to quantify the DNA density on AuNPs. Finally, the particles were resuspended in reaction buffer (10 mM Tris-HCl, 100 mM NaCl, 10 mM $MgCl_2$, 1 mM DTT, pH 7.0) and the L-FS probe concentration was determined as 2 μM.

For Exo III-assisted target recycling amplification: 10 μL L-FS probe-AuNPs was added to 70 μL reaction buffer in a 96-well microplate. Then 10 μL different concentration of target DNA was added to the above microplate and incubated for 10 min at 37 ^{o}C. 10 μL of Exo III (5 unit/uL) was quickly added into the above 96-well microplate format (the finally target DNA concentration was 100 nM, 10 nM, 1 nM) and the fluorescence was recorded immediately by BioTek H1 microplate reader. The background signal was obtained by performing the same reaction with reaction buffer replacyingExo III.

The 15 % PAGE analysis for Exo III-assisted target recycling amplification assay. Three experimental groups and three control groups were set up. For experimental groups: (1) 20 μL target DNA2 (2 μM) and 20 μL L-ssDNA2 (5 μM) were mixed in Exo III reaction buffer (10 mM Tris-HCl, 10 mM $MgCl_2$,1 mM DTT, 100 mM NaCl, pH=7.0) and incubated with boiling water for 5 min. After cooling down to room temperature, 10 μL above solution and 10 μL Exo III (2 unit/μL) were added into 80 μL reaction buffer. (2) 10 μL target DNA2 (1 μM) and 10 μL Exo III (2 unit/μL) were added into 80 μL reaction buffer; (3) 10 μL L-ssDNA probe1 (1 μM) and 10 μL Exo III (2 unit/μL) were added in 80 μL reaction buffer. All mixtures were incubated at 37 ^{o}C for 1 h. The control group was performed with 10 μL reaction buffer instead of Exo III. Finally, the mixtures were investigated by 15% PAGE.

Table S1. The sequence of deoxyribonucleic acid.

Name	Sequence (5′→3′)	Figure
ssDNA1	TTTATATGTTTCTCCTGGAGATAACGCAATCGTGAC AACTTTCGCAAGCGGTG	Figure 1A
ssDNA2	GCTTGCGAAAGTTGTCACGATTGCGTTATCTCCAGG AGAAACATATAAAGGGG	Figure 1A
ssDNA3	CACCGCTTGCGAAAGTTGTCACGATTGCGTTATCTC CAGGAGAAACATATAAA	Figure 1A
L-ssDNA FQ probe 1	FAM-TTTTTTTTTTTT-BHQ1	Figure 1B
90 nt ssDNA	TTTATATGTTTCTCCTGGAGATAACGCAATCGTGAC AACTTTCGCAAGCGGTGTAAGGTAGCAGGCTTCCGA ATTCCGCGTTTTTACGGC	Figure 2,
L-ssDNA FQ probe 2	FAM-ATGTGGAGAA-BHQ1	Figure 3
Target DNA1	CGTACCTGGATTCTCCACATCGTAC	Figure 3
L-FS probe	SH-TTTTTTTTTTGTGTTAGCCTCAAGTG-FAM	Figure 4
Target DNA2	CACTTGAGGCTAACACTTTT	Figure 4
Probe	TTTATATGTTTCTCCTGGAGATAACGCAATCGTGACA ACTTTCGCAAGCGGTG	Figure S1
Target DNA 3	GGGGCACCGCTTGCGAAAGTTGTCACGATTGCGTTA TCTCCAGGAGAAACATATAAAGGGG	Figure S1

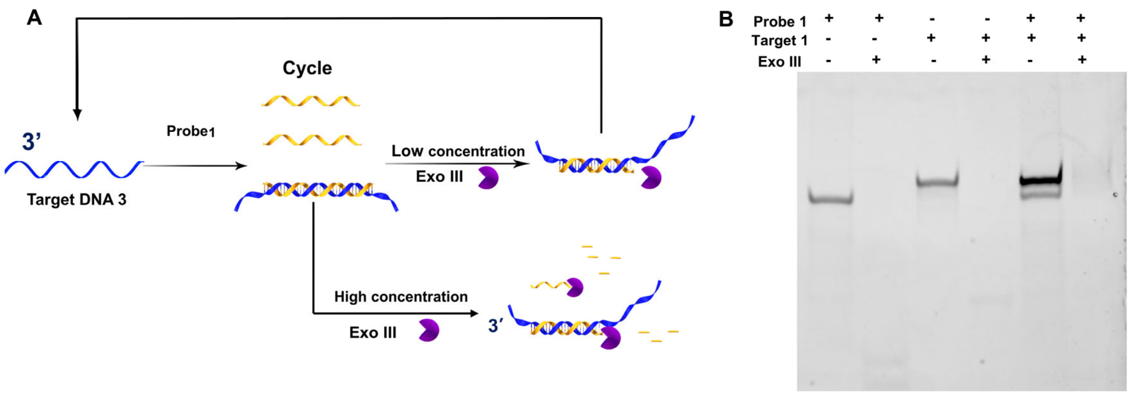

Figure S1. 15% PAGE analysis of Exo III assisted Free L-ssDNA probe in RCA reaction buffer (A) The role of different concentrations of Exo III-assisted target recycling amplification (B) The 15 % PAGE analyzed different concentration target DNA for high concentration of Exo III-assisted target recycling amplification

I.

Reference

1. Qu, X.; Zhu, D.; Yao, G.; Su, S.; Chao, J.; Liu, H.; Zuo, X.; Wang, L.; Shi, J.; Wang, L.; et al. An Exonuclease III-Powered, On-Particle Stochastic DNA Walker. *Angew. Chem.* **2017**, *129*, 1881–1884.
